# Endocrine disrupting chemicals and the growth of environmental health in Israel

**DOI:** 10.1186/2045-4015-1-35

**Published:** 2012-08-28

**Authors:** Henry Falk

**Affiliations:** 1 1571 Nantahalla Court, NE, Atlanta, GA, 30329, USA

## Abstract

This commentary addresses the article by Berman, et al. on reproductive health trends in Israel potentially related to endocrine disrupting chemicals (EDCs) and on associated health policy decisions in Israel to prevent long-term effects from exposure to EDCs. There are intensive, ongoing research efforts in the US that will provide additional guidance on this issue in the future. The commentary also notes and commends the growing capacity and resources for environmental health work in government and academia in Israel.

## 

Surveillance of disease is essential to public health; it is how we know about the many problems we face, both for infectious and chronic diseases. For environmental health though, every case that is counted represents a failure to have anticipated, ascertained, or prevented the causal environmental exposure.

My career at CDC began with the study of fatal liver cancer (Surveillance of disease is essential to public health; it is how we know about the many problems we face, both for infectious and chronic diseases. For environmental health though, every case that is counted represents a failure to have anticipated, ascertained, or prevented the causal environmental exposure.angiosarcoma) in vinyl chloride polymerization workers and of lead poisoning in children. The first of hundreds of cases of liver cancer caused by vinyl chloride was detected in 1974, thirty two years after this production process was introduced and decades too late for tens of thousands of workers already significantly exposed. Similarly, the huge impact of leaded gasoline was noted in the early 1980s, more than a half century after leaded gasoline came into widespread use and generations of children were significantly exposed.

We must look at surveillance data for mortality, incidence of disease, preclinical biologic changes, and associated risk factors; there is much to be learned in this work. At the same time, for assessing risk from chemical exposures, whole scientific disciplines have developed in areas such as hazard identification, exposure analysis, risk characterization, comparative toxicology, and biomonitoring to proactively prevent exposure to toxic chemicals. Because there are many scientific challenges and also because the economic stakes are often very large, the analytic protocols have become very detailed and expensive, and the regulatory processes are often very complex.

Endocrine disrupting chemicals (EDCs) are at the cutting edge of this scientific and public health challenge. There are scores if not hundreds of chemicals to test, often at very low doses. The health effects may be very subtle or the chemicals may only slightly modify the body’s hormonal balance—but of course this may be enough for significant human health effects, especially if one may be exposed to dozens of chemicals possibly having cumulative effects. And much of our historic or standard toxicologic testing regimens, heavily focused on issues like carcinogenesis and teratogenesis, need to be updated.

Berman and colleagues, in this issue of the IJHPR [1], have done a thorough evaluation of the reproductive health data in Israel that one might expect to be affected by EDCs. They have looked at national databases such as the cancer registry, the birth defects surveillance system, and IDF screening examination results, and they have also looked at specific studies done in Israel related to issues such as in vitro fertilization, sperm tests, and specific birth defects. Their conclusion is very carefully stated: the data provide some evidence of adverse reproductive trends, but much of the evidence is limited. This brings us to the heart of the matter: what next? Balancing between the very conservative approach of relying heavily on the Precautionary Principle in the absence of reliable data and the alternative approach of hewing to a very strong evidence base before taking action, the authors have outlined the thoughtful approach being taken in Israel to address this issue. They have also outlined a very appropriate plan of action to continue to collect data in Israel so they can be abreast of any changes occurring within the country. This is all a very well reasoned and careful approach to a difficult challenge and the authors should be commended for their work in this area.

There are efforts underway in the US that will provide helpful data in the future. The EPA has established the Endocrine Disruptor Screening Program [2] to substantially increase the quantity, quality, and timeliness of toxicity testing for EDCs; at the same time, the EPA Office of Research and Development has a strong focus on developing new and expedited testing approaches for EDCs [3], in line with the recommendations of the National Academy of Sciences [4]. The Centers for Disease Control and Prevention (CDC) is conducting extensive biomonitoring for EDCs as part of their nationally representative testing of the US population [5], and other parts of the US government, such as the NIH, also very committed to research in this area.

From the perspective of a distant but interested and engaged colleague, it is very gratifying to see the clear growth in environmental health expertise in Israel exemplified by this article. It is exciting to see a growing organizational locus for such work in the Ministry of Health, as well as new links with scientific investigators in major Israeli universities. This is a real tribute to the leadership in the Ministry in recent years for supporting these endeavors. Certainly, there are many challenging environmental health issues to be addressed in Israel, including air and water pollution and the safe handling of toxic chemicals, and strong programs in government and academia will be needed for sound science and policy development.

Finally, one doesn’t generally focus on the Acknowledgments Section when writing a Commentary or Editorial, but there is something remarkable here: the Environment and Health Fund ( http://www.ehf.org.il/) is cited 4 times. As a member of the technical advisory committee which initially evaluated the need for greater capacity in Israel devoted to environmental health, and as a keen observer of the process over the last 6–7 years, I would like to recognize the foresight and good judgment of Yad Hanadiv in initiating this effort and the disciplined approach and good work of the Environment and Health Fund in implementing this program.

## Competing interests

The author declares that he has no competing interests.

## Author information

Dr. Falk is currently a Consultant in Environmental and Public Health (Falk Consulting). He is a former Director of the National Center for Environmental Health/Agency for Toxic Substances and Disease Registry at the Centers for Disease Control and Prevention (CDC) in Atlanta, GA, and a Rear Admiral (Ret.) in the Commissioned Corps of the US Public Health Service. Currently, he is a Consultant on global health issues to the Deputy Director for Noncommunicable Disease, Injury and Environmental Health at the CDC; he is also an Adjunct Professor of Environmental Health at the Rollins School of Public Health, Emory University.
